# Peripheral interleukin-6-associated microglial QUIN elevation in basolateral amygdala contributed to cognitive dysfunction in a mouse model of postoperative delirium

**DOI:** 10.3389/fmed.2022.998397

**Published:** 2022-09-09

**Authors:** Jing-Lan Mu, Xiao-Dong Liu, Ye-Hong Dong, Ying-Ying Fang, Shi-Da Qiu, Fu Zhang, Ke-Xuan Liu

**Affiliations:** ^1^Department of Anesthesiology, Nanfang Hospital, Southern Medical University, Guangzhou, China; ^2^Department of Anesthesia and Intensive Care, Prince of Wales Hospital, The Chinese University of Hong Kong, Hong Kong, Hong Kong SAR, China; ^3^Peter Hung Pain Research Institute, The Chinese University of Hong Kong, Hong Kong, Hong Kong SAR, China; ^4^Department of Neurobiology, School of Basic Medical Sciences, Southern Medical University, Guangzhou, China

**Keywords:** delirium, repeated intestinal I/R, IL-6, microglia, BLA, QUIN

## Abstract

**Background:**

Developing effective approaches for postoperative delirium has been hampered due to the lack of a pathophysiologically similar animal model to offer insights into the pathogenesis. The study, therefore, aimed to develop a delirium-like mouse model and explore the underlying mechanism.

**Methods:**

The three cycles of 10-min clamp following 5-min reopening of the superior mesenteric artery (SMA) were performed in adult male C57BL/6 mice to induce a delirium-like phenotype. Composite Z score calculated based on the results of Open Field, Y Maze and Buried Food Tests was employed to assess the delirium phenotype in mice. Microglia activities were monitored by immunofluorescence staining and comprehensive morphological analysis. Systemic administration of minocycline (MINO), IL-6 antibody or IL-6 neutralizing antibody, was applied to manipulate microglia. The expressions of Indoleamine 2,3-dioxygenase-1 (IDO-1) and quinolinic acid (QUIN) were examined by RT-PCR and High-Performance Liquid Chromatography/Mass Spectrometry, respectively. Cytokines were measured using fluorescence activated cell sorting method.

**Results:**

The repeated ischemia/reperfusion (I/R) surgery caused significant anxiety (*P* < 0.05) and cognition decline in working memory and orientation (*P* < 0.05) in mice at postoperative 24 h. The composite Z score, indicating an overall disturbance of brain function, fluctuated over 24 h after I/R surgery (*P* < 0.001). Immunofluorescent staining showed that the percentage of microglia in the basolateral amygdala (BLA) (*P* < 0.05) was reactivated after I/R surgery and was negatively correlated with dwell time at Y maze (*R* = −0.759, *P* = 0.035). Inhibiting microglia activities by MINO reduced QUIN productions (*P* < 0.01) that improved cognitive deficits (*P* < 0.05). The peripheral IL-6 might cause IL-6 elevation in the BLA. Systemic administration of IL-6 antibodies suppressed I/R-induced IL-6 elevations (*P* < 0.05), microglial reactivations (*P* < 0.05), IDO-1 expressions (*P* < 0.01), and neuroactive metabolite QUIN productions (*P* < 0.05) in the BLA, resulting in a recovery of cognitive deficits (*P* < 0.05). Injection of IL-6 exerted opposite effects.

**Conclusion:**

The repeated intestinal I/R surgery-induced mouse model is a simple and reproducible one of postoperative delirium. Peripheral IL-6-associated microglial QUIN elevations in the BLA contributed to cognitive dysfunction in the model of postoperative delirium.

## Introduction

Postoperative delirium has been reported to be associated with cognitive decline, higher mortality, and lower quality of life (1, 2). Risk factors for postoperative delirium include acute systemic inflammation, medications (e.g., anticholinergic drugs), and aging-associated neurological abnormalities, such as impaired neuronal network, dysfunction of blood brain barrier (BBB), and reactivation of microglia cells (3).

Resting microglia dynamically surveil the central nervous system (CNS) niches and can be rapidly reactivated when sensing abnormal signals. In this way, microglia play an important role in neuroinflammation, neuropathy, and synaptic strength (4). Reactivated microglia are also known to secrete various bioactive molecules, including inflammatory mediators and neuroactive metabolites (5, 6). The association between microglia and delirium has been demonstrated in both human and animal studies (5, 7, 8). For example, patients with delirium were found to have higher levels of microglia-derived soluble triggering receptor expressed on myeloid cells 2 (TREM2) in cerebrospinal fluid (8). In postmortem tissue sections, it was shown that microglial markers of Human Leukocyte Antigen-DR isotype (HLA-DR) and CD68 were higher in patients with delirium than in age-matched controls (7). In animal studies, microglia reactivation was observed in the hippocampus that dominated neuroinflammation postoperatively. Inhibition of microglial proliferation reversed hippocampal inflammatory mediators, leukocyte invasiveness, and postoperative cognitive decline (5). Despite all these findings, the mediators leading to microglia reactivation and microglia-derived neuroactive substances remain elusive and deserve further exploration in the new studies.

The development of effective treatments for postoperative delirium has been blocked in part due to the lack of a pathophysiologically similar animal model that offers insights into its pathogenesis. Several animal models of delirium have been developed, but there are limitations with these models. Systemic LPS administration is a popular approach to induce behavioral disturbances in animals to mimic a delirium-like state (9–11). However, LPS challenge is not a clinically established factor for delirium, in addition, most of these studies used a single behavioral test to assess such a complicated disease. Small intestine I/R-induced animal model could alter brain function from the short to long term (12–14). However, the models involve long-time ischemic attack (usually 60–90 min), resulting in high immediately postoperative mortality and prolonged neurological abnormalities that are inconsistent with features of clinical postoperative delirium. Recently, Peng et al. (15) reported that a simple laparotomy induced delirium-related behaviors. Furthermore, they developed a scoring tool analogous to Confusion Assessment Method (CAM) algorithm (diagnosis tool for clinical delirium) (16) to assess the delirium-like phenotypes in rodents based on the outcomes of all behavior tests. This model included a simple midline abdominal incision under isoflurane anesthesia without affecting any intra-abdominal organs.

In this study, we attempt to improve the mouse model of postoperative delirium by applying laparotomy and attacks through three cycles of transient ischemia (10 min) and reperfusion (5 min). This repeated protocol was devised from the recognition that intermittent ischemia potentiates intestinal reperfusion injury (17). To comprehensively assess the delirium-like phenotype, composite Z scores were calculated based on a battery of behavior tests, namely Open Field Test, Y Maze Test, and Buried Food Test. Finally, microglia reactivation induced by peripheral IL-6 in the associated brain region, and neuroactive molecule of QUIN were found and studied using this new mouse model of postoperative delirium.

## Materials and methods

### Animals

All animal procedures were approved by the Animal Experiment and Welfare Committee of Nanfang Hospital, Southern Medical University (Guangzhou, China). Male C57BL/6 mouse (male, 8 weeks old, 20–22 g) were obtained from SPF Biotechnology (Beijing, China). Mice were housed in a controlled environment (temperature 20–22°C; humidity of 50 ± 10%; 12 h of light/dark on a reversed cycle) with *ad libitum* access to food and water. All mice were acclimated to the environment for 7 days before performing behavioral tests between 12:00 PM and 5:00 PM on the experimental day. Researchers who were blinded to the grouping conducted behavioral experiments and data collection.

### Surgery and experimental protocol

The intestinal ischemia and reperfusion surgery was performed as described previously (12). Animals were fasted 12 h before surgery. Under isoflurane anesthesia, the small intestine was exteriorized through a 1-cm midline abdominal incision. The SMA was clamped for 10 min using a microvascular clip (18055-02, 85 g, F.T.S., CA, United States) following revascularization for 5 min. This cycle was repeated three times before closing the wound. Cream containing 2.5% lidocaine was applied immediately and every 8 h to treat incisional pain. Animals at sham group underwent all procedures except for SAM clamping and revascularization.

The postoperative delirium-like phenotypes of mice were assessed according to previous methods developed by Peng et al. (15) with minor modifications (Table 1). Briefly, all mice were evaluated in a battery of neurological tests, namely Open Field Test, Y Maze Test, and Buried Food Test 7 days before surgery (baseline) and 6, 9, 24 h after operation. Latency to eat (Buried Food Test), time spent in the center (Open Field Test), latency to the center (Open Field Test), freeze time (Open Field Test), number of entries in novel arm (Y Maze Test), duration in novel arm (Y Maze Test), and first choice of novel arm (Y Maze Test) were then extracted to compare changes in neurological activities between groups and used to calculate composite Z scores to monitor delirium-like manifestation between treatment or over time.

**TABLE 1:** The tool to assess postoperative delirium for mice.

1. Acute onset and fluctuating course: Mean composite Z score fluctuates during postoperative 24 h
2. Inattention: Buried food test/open field test/Y maze
3. Disorganized thinking: Buried food test/open field test/Y maze
4. Altered level of consciousness: Buried food test/open field test/Y maze
**1 + 2/3/4 abnormity:** Delirium-like phenotype

**FIGURE 1:**
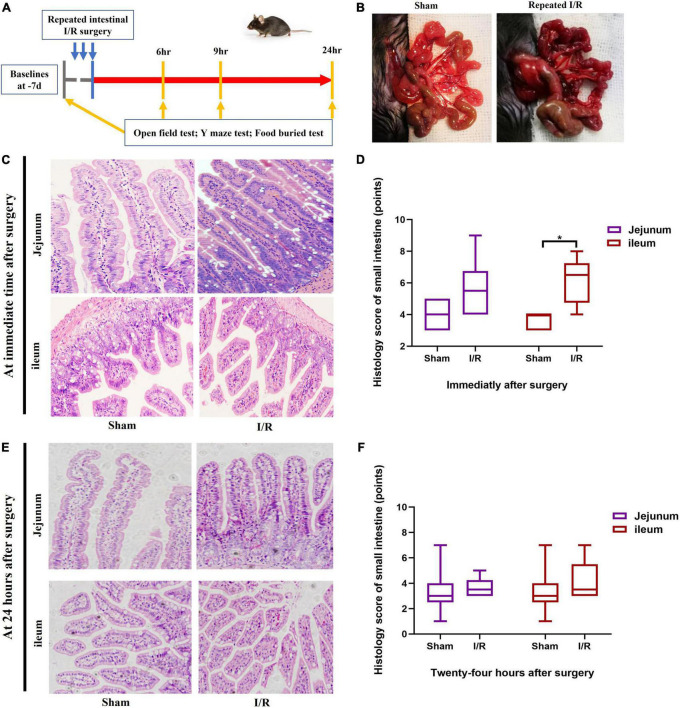
Experimental protocol and histological assessment on small intestine. **(A)** Experimental protocol. **(B)** Small intestine changed due to I/R or sham operation. **(C,D)** Histological assessment on small intestine immediately after surgery. **(E,F)** Histological assessment on small intestine at postoperative 24 h. *N* = 10 per group for behavioral testing, *N* = 8 per group for histological assessment, *P < 0.05. I/R: ischemia and reperfusion, d: days, hr: hours.

The experimental protocol is illustrated in Figure 1A. Initial microglia assessment across whole brain was detected at 6, 9, 24 h after surgery as well. Histologic examination of small intestine was performed at immediate time and the severest brain disturbance time after surgery. Pro-inflammatory cytokines of IL-1β, IL-6, and TNF-α, suggested by results of clinical trials for delirium (18), were measured along the intestine-brain axis at the severest disturbance time. We explored underlying mechanism for core symptom of cognitive dysfunction, detected by Y maze, because it is a such complicated disease with a constellation of symptoms.

### Intervention

Minocycline (MINO, Sigma-Aldrich M9511, St. Louis, MO, United States) was employed to inhibit microglia activation. MINO was dissolved in normal saline and injected intraperitoneally at 50 mg/kg 1.5 h before behavioral experiments (19). Recombinant IL-6 (PeproTech 200-06, Cranbury, NJ, United States) and mouse IL-6 neutralizing antibody (R&D Systems, Minneapolis, MN, United States) were given to study the role that peripheral IL-6 played in this model. IL-6 of 50 μg/kg were i.p injected at immediate, 6 and 12 h (20, 21). Anti-IL-6 antibody of 0.1 μg/mouse was injected via tail vein at postoperative 6 h when the first behavioral testing was performed (13). The large size of IL-6 antibody and its complex with IL-6 (> 150 kD) makes them impossible to cross the blood-brain barrier to affect the brain directly (22, 23).

### Open Field Test

Open Field Test was performed as previous studies (13, 15). Mice were moved into the experimental room 1 h prior to the testing. A mouse was then gently placed in the center of an open field and allowed to freely explore the chamber for a period of 10 min. Total distance, rearing time, and duration at the border and in the center were recorded by VersaMax (v3.02-125, Omnitech Electronics, Columbus, OH, United States).

### Y Maze Test

Y Maze Test was used to assess cognition-associated spatial memory and orientation (24). The maze is comprised of three arms (8 × 30 × 15 cm, width × length × height), with an angle of 120 degrees between each arm. In the study, the recognition memory protocol was implemented including two trials (15, 24). During the first training trial, one arm of the Y maze was blocked, and a mouse was allowed to freely explore the starting arm and the other arm for 10 min. After 1 h at the second trial with all three arms accessible, the mouse was returned to the maze for a 5-min exploration to test the memory recognition. The starting arm and the other arm had been randomly set to avoid potential bias. A video camera linked to EthoVision (v7.0, Noldus, Wageningen, Netherlands) was installed 60 centimeters above the maze to monitor and analyze number of arm entries, the time spent in each arm, and the first choice of the arm.

### Buried Food Test

Buried Food Test was performed to examine the latency to forage food buried in the cage bedding (15, 25). Mice were familiarized with cereals for 2 days before the experiments. On the experimental day, the mice were acclimated to the testing room for an hour prior. A cereal pellet was buried 0.5 centimeters below surface of bedding (3 cm high) and was located randomly at each time. A mouse was placed in the center of the testing cage and allowed to search for the cereal pellet for 5 min. When the mouse finding out the food and grasping it with forepaws and/or teeth, we recorded the time latency and stopped the experiment. If a mouse failed to find the food over a period of 5 min, the latency was recorded as 300 s. The clean cage, fresh bedding, new gloves, and new pellet of cereal were used for each mouse to prevent transmission of olfactory cues.

### Hematoxylin and eosin staining and scoring

Jejunum and ileum were harvested and stained with H&E for histologic examination. The histologic scoring scale (26) was applied to assess the tissue injury caused by ischemia and reperfusion. The scoring system includes three categories relating to mucosal damage, inflammation, and hyperemia/hemorrhage, each with 0–5 points (normal to severe) (Supplementary Table 1).

### Immunofluorescence and microglial analysis

The animals were transcardially perfused with 4% paraformaldehyde. The brains were taken and fixed in paraformaldehyde for 4–6 h, followed by cryoprotection with 30% sucrose and the organs were cut into 40 μm sections. For microglial detection, the sections were incubated with rabbit-anti IBA-1 (1:500; Wako Pure Chemicals, Osaka, Japan) and further with fluorescein isothiocyanate-conjugated secondary antibody (1:500; goat anti-rabbit, Invitrogen, Waltham, MA, United States). Images of brain were acquired using LSM880 confocal microscope (Carl Zeiss, Oberkochen, Germany) controlled by Zen2010 software (Carl Zeiss, Jena, Germany). Regions of plaques were pictured at 400 × magnifications. Images were sampled at a resolution of 1024*1024 pixels. A multi-plane Z mode allowed to capture 20 images (2 μm thick) in 40 μm depth of the tissue section, which were later combined to obtain a single high-quality confocal image.

Microglia activation was initially assessed by phenotypic characterization of stages one to five cells (4, 27). Microglia at stages from three to five with cell body increasing and processes shrinking, were considered reactivation (4, 27). All Iba-1 positive cells were graded twice in an area of 0.09 mm^2^ using image analysis software (Image J, v1.52a, NIH, United States). The percentage of reactivated microglia was measured in the basolateral amygdala (BLA) (28), medial prefrontal cortex (PFC) Zilles cg1, Cornu Ammonis (CA1), Cornu Ammonia 3 (CA3), Dentate Gyrus inner blade (DGib), and striatum (STR), which are well recognized to be associated with emotions and cognition (29–31). In the mechanism studies, a comprehensive analysis of microglia was performed on 3D images without projection. Imaris (v9.0.1, Bit plane, Belfast, United Kingdom) was applied to create surface and trace filaments of microglia, followed by quantification of cell number, cell volume, cumulative process length, and number of branch points in these images. The process included the following steps: (1) segment microglia from background and mark the cells with somas, processes, and branch points, and use the same Gama value for all images, (2) create a skeleton to represent the 3D structure of cell body and processes, (3) quantify the microglia (32, 33).

### Quantification of pro-inflammatory cytokines

The concentrations of IL-1β, IL-6, and TNF-α were determined using the BD Mouse/Rat Soluble Protein Master Buffer Kit (558266, BD Biosciences, San Jose, CA, United States) in conjunction with the BD CBA Flex Sets for the specific detection of mouse IL-1β (560232), IL-6 (558301), and TNF-α (558299). The assays were conducted and analyzed according to the manufacturer’s instructions. Briefly, 50 μl of serially diluted standards or test samples were incubated with 50 μl of capture beads and then with 50 μl of PE detection reagent, followed by quantification of cytokine concentrations using the BD LSRFortessa™ X-20 flow cytometer (BD Bioscience) and FCAP array software (version 3.0, BD Biosciences).

### RT-PCR for IDO-1 mRNA in basolateral amygdala

Mouse was transcardially perfused with normal saline to wash out blood. The brain was quickly removed from the skull and was chilled on ice for 10 min. Bilateral dissections of the BLA regions were performed according to a previous report (34). Coronal sections between Bregma −1 and Bregma −2.75 were isolated and verified under microscope. The sections were placed caudal side up. Cuts were then made in the lower-left and lower-right corners while avoiding any hippocampal tissue. Isolated BLA tissues were placed in the tube containing TRIzol reagent (Invitrogen, New York, NY, United States) and homogenized for 120 s. Total RNA was then extracted using chloroform and precipitated with isopropanol, followed by conversion to complementary DNA (cDNA) using SYBR Green kit (TOYOBO, Tokyo, Japan). The following primers were used: forward 5′-TGCCTCCTATTCTGTCTTATGC-3′ and reverse 5′-CTTTCAGGTCTTGACGCTCTAC-3′. PCR was performed using the ABI Q5 Real-Time PCR System (Applied Biosystems, Foster City, CA, United States). The relative IDO-1 gene expression was determined by normalization to the housekeeping gene (β-actin) and the IDO-1 gene in the control group using the 2−ΔΔ CT method.

### Quantification of QUIN using high-performance liquid chromatography/mass spectrometry

Standards were dissolved in caffeic acid (Macklin, Shanghai, China) solution. Internal standards (IS) were added to each standard and sample for a final concentration of 10 ng/ml to correct for sample and instrument variability. Tissue homogenizations (50 μl) were diluted 12-fold (w/v) by adding 10 μl IS, 500 μl water and 140 μl acetonitrile. Diluted samples were then filtered through Amicon Ultrafilter (Millipore, Billerica, MA, United States) by centrifugation at 15,000 *g* for 10 min at 4°C. Quantifications of QUIN were determined by HPLC (Waters, Milford, MA, United States) with tandem mass spectrometry (MS/MS) (Thermo Scientific, Waltham, MA, United States). Samples were run in positive ionization mode optimized for QUIN detection. Resultant acquisitions were directly injected into the Waters, equipped with an C18 (waters T3, 2.1 × 100 mm, 1.7 μm) column. The mobile phase consisted of an aqueous component (A: 10 mM ammonium acetate + 0.1% formic acid in ultrapure water) and an organic component (B: 0.1% formic acid in acetonitrile). The elution gradient was used as follows: 100% A for 30 s and 90% B for 6 min. The flow rate was set at 0.25 ml/min and the run time for each sample was 13 min. The concentration of QUIN in each sample was quantified by comparison to the standard curve.

### Statistical analysis

Variables were presented as mean (standard deviation, SD) or median (interquartile range, IQR). The behavior parameters at postoperative 6, 9, and 24 h were presented as percentages compared to their baselines. An independent Student’s *t*-test or Mann–Whitney *U* test was used to compare results where appropriate. Fisher’s exact test chi-square was used to test first choice of the novel arm at Y maze. Z score was calculated using formula described by Moller et al. (35) and Peng et al. (15). *Z* = ΔX-I/R – MEAN (ΔX-sham)/SD (ΔX-sham). ΔX-sham was the change score of mice in sham group at 6, 9, and 24 h after operation minus the baseline score; ΔX-I/R was the change score of mice in I/R group at 6, 9, and 24 h minus corresponding baseline score; MEAN (ΔX-sham) was the mean of ΔX-sham; SD (ΔX-sham) was the standard deviation of ΔX-sham. The composite Z score was calculated as sum of six Z scores (latency to eat food, time spent in the center, latency to the center, rearing time, entries in novel arm, and duration in novel arm) normalized with SD for that sum. Spearman’s rank correlation was used to analyze correlation analysis between the cognitive outcomes of Y Maze Test and the percentages of reactivated microglia in each brain region. The one-way analysis of variance or the Kruskal–Wallis test was used for comparing three groups depending on the nature of data. Statistical analysis was performed in SPSS (version 23.0; SPSS for Windows, Chicago, IL, United States) and Graph Pad Prism 8.0 (GraphPad Software Inc., United States). A two-tailed *P* < 0.05 was set as statistical significance.

## Results

### Repeated I/R insults in the small intestine of mice mimicked the transient and reversible I/R injury and delirium associated with abdominal surgery

The timeline of the experimental design was shown in Figure 1A. I/R injury was introduced by performing three cycles of 10-min clamping and 5-min reopening of the SMA. The small intestine of I/R mice turned from red to dark purple and became distended after completing surgery (Figure 1B). The jejunum and ileum were collected to assess histological injury using H&E staining. The histological score of ileum, representing severities of inflammation and hyperemia, was higher in I/R mice than the score of sham mice at immediate time after surgery (*P* = 0.020, Figures 1C,D). There was no difference in jejunal score between groups (*P* = 0.097, Figures 1C,D). At 24 h postoperatively, the histological scores of both ileum and jejunum were similar between the two groups (*P* > 0.05 for both, Figures 1E,F). This indicated that the injuries caused by this repeated I/R interventions were transient and reversible, similar to the nature of I/R injury during abdominal surgery and the associated delirium. There was no death occurred during the experimental period.

### Repeated I/R insult in the small intestine resulted in a delirium-like manifestation in the mouse

**FIGURE 2:**
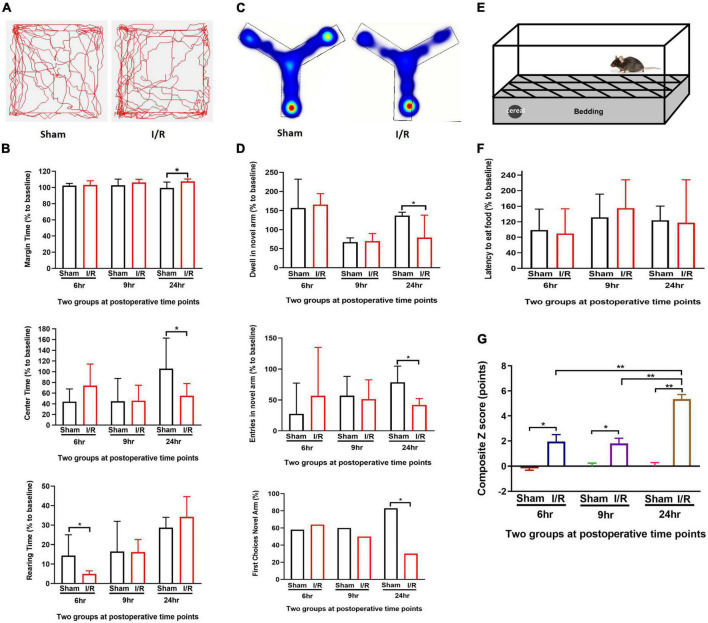
Repeated I/R surgery of small intestine disturbed the behaviors in mice at postoperative 6, 9, and 24 h which had higher composite Z scores. **(A)** Open Field Test. **(B)** Y Maze Test. **(C)** Buried Food Test. **(D)** Track map of Open Field Test. **(E)** Heating map of Y Maze Test. **(F)** Composite Z score of each mouse. **(G)** Summary of composite Z scores. The percentage data were presented as median (IQR). Composite Z score was presented as Mean ± SD. Larger values of composite Z score suggest severer impairment. *N* = 10 per group, *P < 0.05, **P < 0.01. I/R: ischemia and reperfusion, hr: hours.

We investigated whether mice could develop delirium-like manifestation by assessing behavioral changes in Open Feld test, Y Maze Test, and Buried Food test. At Open Feld test, I/R insult increased the marginal time while decreased the center time in the mice compared to those of the sham at 24 h postoperatively (marginal time: 107.50 vs. 99.39%, *P* = 0.022; center time: 55.11 vs. 105.80%, *P* = 0.020, Figures 2A,B), The results suggested anxiety induced by the intestinal I/R surgery. The I/R mice spent shorter rearing time than the sham at 6 h (4.86 vs. 14.31%, *P* = 0.007, Figure 2B), but not 9 and 24 h postoperatively (both *P* > 0.05, Figure 2B), indicating I/R injury suppress the willingness to explore in the early time. The I/R surgery did not alter the total distance between the two groups at any time point (Supplementary Table 2). At Y maze testing, I/R insult reduced duration and number of entries in the novel arm as compared to the sham at postoperative 24 h (durations: 75 vs. 101%, *P* = 0.036, entries: 41.88 vs. 78.53%, *P* = 0.002, Figures 2C,D), indicating the impaired working memory and spatial orientation in mice with intestinal I/R injury. Consistent with this result, fewer percent of I/R-injured mice initially chose the novel arm for entry than the sham-operated mice at the same time point (30 vs. 83%, *P* = 0.027, Figure 2D), indicating that the I/R surgery reduced interest in exploration in those mice. We did not find any difference in the arm visits between the two groups either (Supplementary Table 3). At Buried Food testing, the repeated I/R insult did not change the latency to eat the food (*P* > 0.05, Figures 2E,F). Composite Z score for each mouse was calculated based on the three behavioral tests (Table 2 and Figure 2G). A higher score indicates worse performance. We noted that the composite Z score of I/R mice fluctuated over 24 h after surgery and peaked at 24 h (5.36 vs. 1.96, 5.36 vs. 1.80, both *P* < 0.001, Table 2 and Figure 2G). In addition, mice undergoing I/R surgery were graded the higher Z scores than the sham at all assessed time points (6 h: 1.96 vs. −0.17, *P* = 0.004; 9 h: 1.80 vs. 0.000, *P* = 0.001; 24 h: 5.36 vs. 0.000, *P* < 0.001, Table 2 and Figure 2G). These results showed that repeated I/R insults in the small intestine could successfully develop a manifestation with the feature of postoperative delirium, which shows an acute and fluctuating change in mental level.

**TABLE 2:** Summary of composite Z scores in sham and I/R mice.

	Z scores at each time point		
	
Mouse	6 h	9 h	24 h
Sham 1	−0.47	−0.32	0.90
Sham 2	−0.60	−0.39	−0.10
Sham 3	0.24	0.45	−1.58
Sham 4	0.26	−0.87	1.40
Sham 5	0.22	−0.88	−0.11
Sham 6	−0.77	1.13	−0.63
Sham 7	0.24	0.89	−0.23
Sham 8	0.21	−0.22	−0.41
Sham 9	−0.21	−0.64	−0.20
Sham 10	−0.81	0.85	0.96
Sham mean	−0.17	0.00	0.00
Sham SD	0.14	0.25	0.28
**Comparison of the scores at the three time points**			>0.05
I/R 1	2.11	2.59	3.55
I/R 2	0.90	2.74	3.37
I/R 3	0.18	3.57	7.07
I/R 4	2.91	2.10	5.36
I/R 5	−1.10	−1.28	6.08
I/R 6	2.72	2.28	5.52
I/R 7	1.31	1.04	5.28
I/R 8	5.33	1.56	6.08
I/R 9	2.50	1.31	5.21
I/R 10	2.69	2.12	6.10
I/R mean	1.96	1.80	5.36
I/R SD	0.56	0.41	0.36
**Comparison of the scores at the three time points**			<0.001**
**Comparison of the scores between sham and I/R mice at each time point**	0.004**	0.001**	<0.001**

**P < 0.01. I/R, ischemia and reperfusion; hr: hours.

### Transient intestinal I/R injury-induced microglial reactivation in the basolateral amygdala that was negatively correlated with delirium-associated cognitive dysfunction and the expressions of cytokines

**FIGURE 3:**
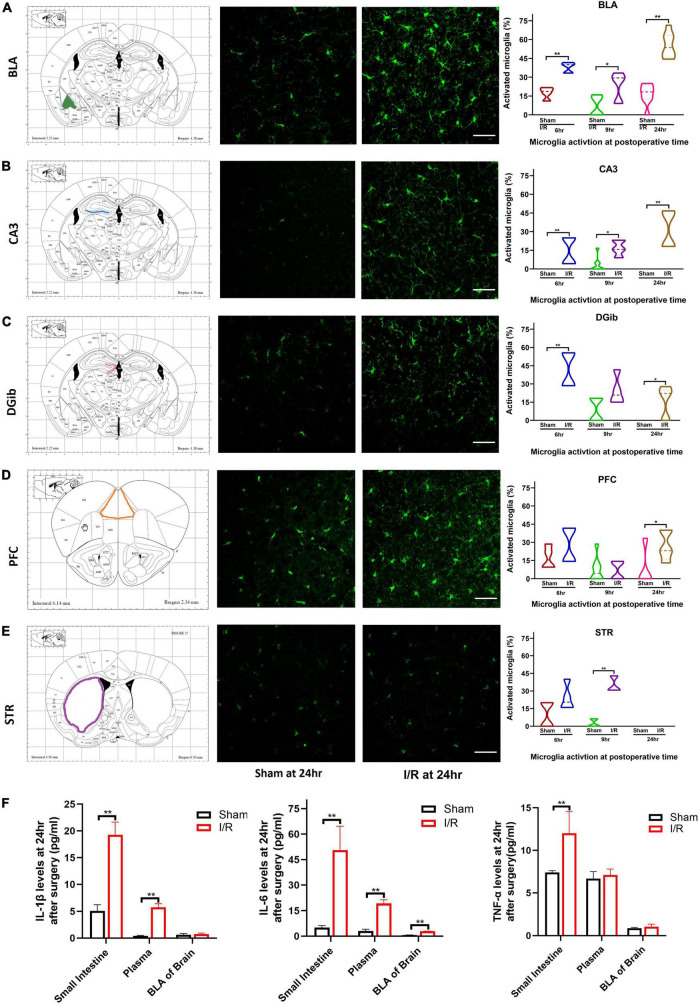
Microglia reactivation in different brain regions and levels of proinflammatory cytokines at 24 h after surgery. **(A)** BLA. **(B)** CA3. **(C)** DGib. **(D)** PFC. **(E)** STR. **(F)** Levels of IL-1β, IL-6, TNF-α in intestine, plasma, and BLA. The percentage data were expressed as median (IQR). *N* = 8 per group, *P < 0.05, **P < 0.01. I/R, ischemia and reperfusion; hr, hours; BLA, basolateral amygdala; CA3, Cornu Ammonis; DGib, dentate gyrus inner blade; PFC, medial prefrontal cortex; STR, striatum.

Microglia can sense and rapidly adapt to locally environmental changes. We examined microglia reactivation in different brain regions associated with delirium (Figures 3A–E). For BLA and CA3, it was observed that the percentage of microglia activated was higher in I/R mice than that of the sham at 6 h (BLA: 40.00 vs. 18.62%, *P* < 0.01; CA3: 14.67 vs. 0%, *P* < 0.01, Figures 3A,B), 9 h (BLA: 29.41 vs. 6.45%, *P* = 0.029; CA3: 15.79 vs. 0%, *P* < 0.01, Figures 3A,B), and 24 h (BLA: 53.57 vs. 18.33%, *P* < 0.01; CA3: 31.31 vs. 0%, *P* < 0.01, Figures 3A,B). For DGib of hippocampus, the percentage of microglia activated was greater in I/R mice at 6 h (42.06 vs. 0%, *P* < 0.01, Figure 3C) and 24 h (22.22 vs. 0%, *P* = 0.026, Figure 3C), but not at 9 h (*P* = 0.111, Figure 3C). In PFC, we only found the higher value in I/R mice at 24 h (23.08 vs. 0%, *P* = 0.016, Figure 3D), while in STR we observed significant microglia activation only at postoperative 9 h (30.77 vs. 0%, *P* < 0.01, Figure 3E). There were no microglia activated found in CA1 of hippocampus (data not shown).

Cognitive dysfunction underpins postoperative delirium. We further examined the brain regions involved in cognitive dysfunction 24 h after surgery by performing correlation analyses between behavioral changes in the Y Maze Test and the percentage of activated microglia in each brain region. It was only found that a negative correlation between dwell time in the novel arm and the percentage of activated microglia in BLA in I/R mice (*R* = −0.759, *P* = 0.035, Table 3), suggesting that abnormal microglia activation in BLA may play a role in postoperative delirium. We thus chose brain BLA region to initially investigate the underlying mechanism for such a complicated brain disorder.

**TABLE 3:** The correlation between the microglia activation and quantitative outcomes of Y maze at 24 h in the I/R mice.

Brain regions Outcomes	BLA	CA3	DGib	PFC	STR
Dwell in novel arm	−0.759*	−0.433	−0.546	−0.609	nil
Entries in novel arm	−0.633	−0.636	−0.403	−0.685	nil

*P < 0.05. I/R, ischemia and reperfusion; BLA, basolateral amygdala; CA3, Cornu Ammonis; DGib, dentate gyrus inner blade; PFC, medial prefrontal cortex; STR, striatum; nil, non-existent.

The up-regulation of IL-1β, IL-6, and TNF-α in the postoperative period have been reported by extensive studies. In this model, expression of IL-6 was elevated remarkably in all three samples compared with those of the sham (*P* < 0.01, Figure 3F). Production of IL-1β of I/R mice was increased in the small intestine and plasma but not in the BLA compared with the sham (both *P* < 0.01, *P* > 0.05, Figure 3F). Production of TNF-α was only higher in the small intestine of I/R mice than the sham (*P* < 0.01, Figure 3F). The results indicated that proinflammatory IL-6 persisted highly in the small intestine, blood, and BLA in I/R-induce delirium-like mice.

### Inhibition of microglial activation and associated IDO-1/QUIN in basolateral amygdala by minocycline led to cognitive improvement in I/R-induced mice

**FIGURE 4:**
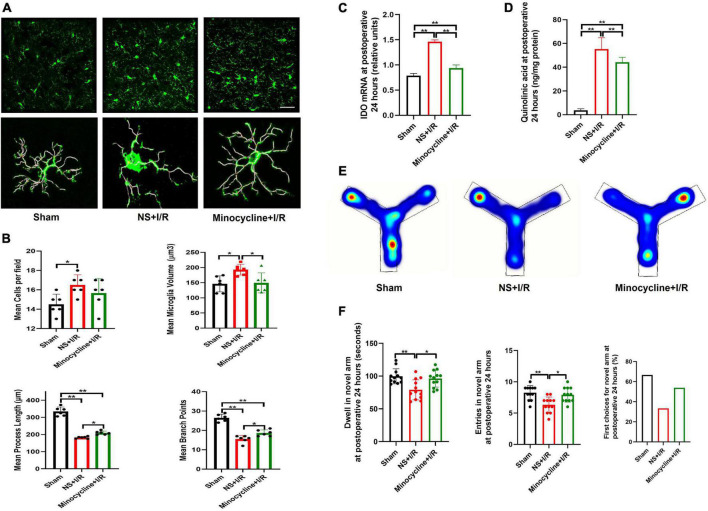
Microglia reactivation and IDO-1/QUIN in BLA inhibited by MINO improved the cognition in the model mice. **(A)** Immunofluorescence staining and skeleton of microglia. **(B)** Analysis of microglia reactivation. **(C)** IDO-1 mRNA expression in BLA. **(D)** QUIN levels in BLA. **(E)** Heating map of Y Maze Test. **(F)** Results of Y Maze Test. *N* = 10–12 per group for Y maze testing, *N* = 8 for others, *P < 0.05, **P < 0.01. I/R, ischemia and reperfusion; BLA, basolateral amygdala; NS, normal saline; MINO, minocycline.

To further assess microglial involvement, the widely used microglia inhibitor MINO was administered to the I/R-injured mice. At postoperative 24 h, microglia activation in BLA was found to be attenuated in MINO-injected mice, as reflected in smaller microglia volumes (*P* = 0.031, Figures 4A,B), longer process lengths (*P* = 0.029, Figures 4A,B), and more branch points (*P* = 0.021, Figures 4A,B) compared to vehicle administration, although the number of microglia was not affected (*P* > 0.05, Figure 4B). We then assessed the expressions of IDO-1 mRNA (the rate-limiting metabolic enzyme of the kynurenine pathway) Ana QUIN (endogenous neuroexcitatory metabolite of kynurenine pathway) in the BLA. IDO-1 mRNA amplifications and QUIN levels were lower in MINO-injected mice than vehicle-injected mice (*P* < 0.01, Figures 4C,D). Consistent with the improvements in biochemistries, MINO also rescued the cognitive impairment caused by I/R insult, indicated by longer dwell time (*P* = 0.025, Figures 4E,F), more entries in the novel arm (*P* = 0.027, Figures 4E,F) at Y maze testing, and propensity to choose the novel arm for the first entry (*P* > 0.05, Figure 4F).

### Peripheral IL-6 promoted microglial QUIN production in basolateral amygdala and exacerbated cognitive impairment in I/R-injured mice

**FIGURE 5:**
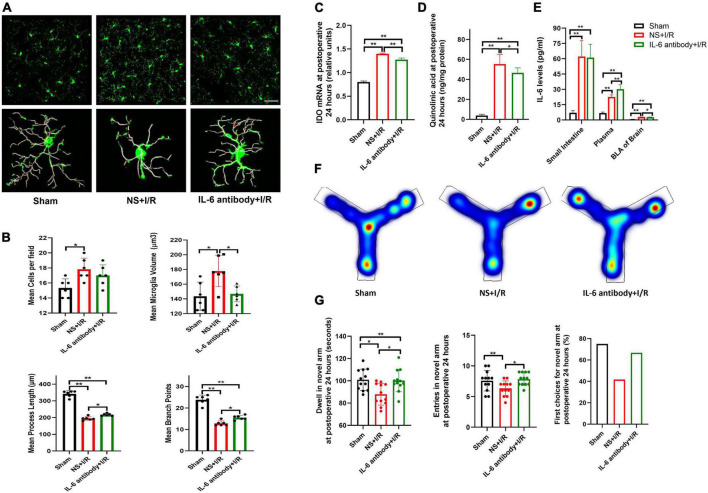
Neutralizing peripheral IL-6 reduced microglia reactivation and IDO-1/QUIN in BLA that improved the cognition in the model mice. **(A)** Immunofluorescence staining and skeleton of microglia. **(B)** Analysis of microglia reactivation. **(C)** IDO-1 mRNA expression in BLA. **(D)** QUIN levels in BLA. **(E)** IL-6 levels in intestine, plasma and BLA. **(F)** Heating map of Y Maze Test. **(G)** Results of Y Maze Test. *N* = 10–12 per group for Y maze testing, *N* = 8 for others, *P < 0.05, **P < 0.01. I/R, ischemia and reperfusion; BLA, basolateral amygdala; NS, normal saline; IL-6 antibody, Interleukin 6 antibody.

As shown above, intestinal I/R injury resulted in dramatic up-regulation of IL-6 in the intestine and blood. We next examined whether manipulation of peripheral IL-6 affected injury-related microglial reactivation and cognitive impairment. Compared with vehicle group, intravenous injection of IL-6 antibody inhibited microglia activation in the BLA in I/R mice, indicated by smaller microglia volumes (*P* = 0.021, Figures 5A,B), longer process lengths (*P* = 0.028, Figures 5A,B), and fewer branch points (*P* = 0.022, Figures 5A,B), but numbers of cells did not change (*P* > 0.05, Figures 5A,B). In parallel, the IDO-1 mRNA syntheses and QUIN productions in the BLA were also reduced in I/R mice after IL-6 antibody administration (*P* = 0.029 for I/R vs. I/R + IL-6 antibody; *P* < 0.01 for the rest, Figures 5C,D). At 24 h after surgery, IL-6 neutralizing antibody did not affect IL-6 expressions in the intestine (*P* > 0.05, Figure 5E), but increased the plasma levels of IL-6 and IL-6 antibody complex (*P* < 0.01, Figure 5E). As a result of treatment, IL-6 neutralizing antibody significantly decreased IL-6 levels in BLA (*P* = 0.033, Figure 5E). The performances of IL-6 antibody-injected I/R mice at Y maze testing were improved in dwelling time and entries in the novel arm they never explored before (*P* = 0.031, *P* = 0.033, Figures 5F,G).

**FIGURE 6:**
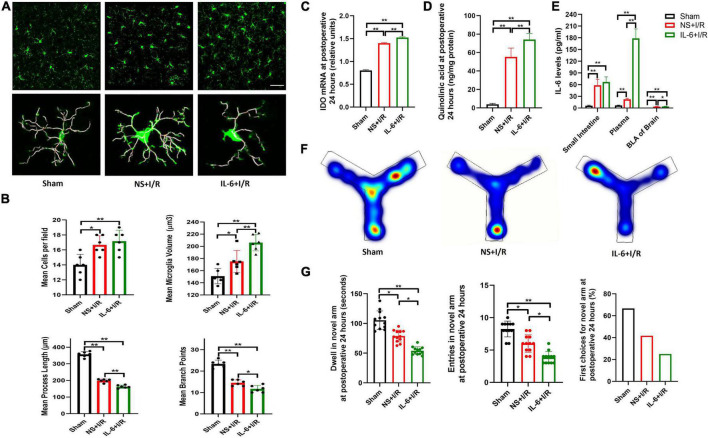
Microglia reactivation and productions of IDO-1/QUIN in BLA triggered by extra peripheral IL-6 worsened the cognition in model mice. **(A)** Immunofluorescence staining and skeleton of microglia. **(B)** Analysis of microglia reactivation. **(C)** IDO-1 mRNA expression in BLA. **(D)** QUIN levels in BLA. **(E)** IL-6 levels in intestine, plasma and BLA. **(F)** Heating map of Y Maze Test. **(G)** Results of Y Maze Test. *N* = 10–12 per group for Y maze testing, *N* = 8 for others, *P < 0.05, **P < 0.01. I/R, ischemia and reperfusion; BLA, basolateral amygdala; NS, normal saline; IL-6, Interleukin 6.

To examine the role of elevated IL-6, we injected IL-6 or vehicle three times after I/R surgery via intraperitoneal injection. Additional IL-6 did not promote microglia proliferation in I/R mice (*P* > 0.05, Figures 6A,B). Compared to vehicle, IL-6 protein further activated microglia, as evidenced by increased microglia volumes (*P* < 0.01 Figures 6A,B), decreased process lengths (*P* < 0.001, Figures 6A,B), and fewer branch points (*P* = 0.020, Figures 6A,B). Microglia cells were not proliferated by extra peripheral IL-6 at postoperative 24 h (*P* > 0.05, Figure 6B). In the meantime, I/R injury-induced upregulation of IDO-1 mRNA and QUIN in BLA were significantly increased by IL-6 treatment (*P* < 0.01, Figures 6C,D). The blood and BLA pure IL-6 levels were significantly elevated at 24 h (*P* < 0.01, *P* = 0.020, Figure 6E) but not in intestine (*P* > 0.05, Figure 6E). As a result of elevated peripheral IL-6, the mice performed worst at Y maze indicated by the shortest stay and least enters in the novel arm (*P* = 0.014, *P* = 0.031, Figures 6F,G). Overall, the results suggested that peripheral IL-6 contributed to CNS neuroinflammation and cognition impairment in delirium.

### QUIN levels of basolateral amygdala were negatively correlated with dwell time at Y maze in the I/R mice

**FIGURE 7:**
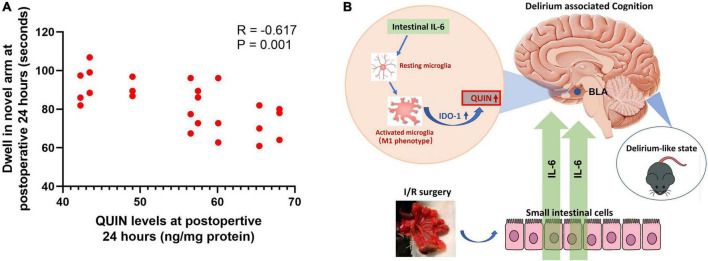
Correlation analysis between QUIN and dwell time at Y maze and illustration of the pathway mediated the delirium-associated cognition in the model. **(A)** Correlation analysis in the I/R mice model, *N* = 24 per group. **(B)** Illustration of the pathway.

Analysis was conducted using the I/R mice which both measured QUIN and performed Y maze testing (*n* = 24). Results showed that higher QUIN levels of BLA were correlated with longer dwell time in the novel arm at Y maze (*R* = −0.617, *P* = 0.001, Figure 7A). The analysis supported the QUIN levels associated with on delirious cognition in the model. In summary, as illustrated in Figure 7B, peripheral IL-6 mediating microglia activation in the BLA of brain, which promoted IDO-1 mRNA amplification and its catabolite QUIN production, eventually introduced the delirium-like state in I/R mice.

## Discussion

The current study reported a simple and reproducible mouse model of postoperative delirium by introducing three cycles of transient I/R attacks into the SMA. The model rapidly developed delirium-associated symptoms, including anxiety and cognitive impairment, within 24 h after surgery, mimicking the manifestation of postoperative delirium in clinical practice. Using this model, we reported that rapid BLA modulation may be implicated in postoperative delirium. We found that microglia reactivation accompanied by upregulation of IDO-1 mRNA and its neuroexcitatory metabolite QUIN in BLA was associated with postoperative cognitive impairment. Furthermore, we revealed that elevated peripheral IL-6 contributed to upregulated levels of IL-6, reactivated microglia, and microglial IDO-1/QUIN in BLA after surgery. Our study provided new insights into the neurological basis of postoperative delirium and highlighted Il-6, microglia, and QUIN as targets for the treatment of postoperative delirium.

Abdominal aortic aneurysm open repair (AAA) is a surgical procedure that involves occlusion of mesenteric arteries and mesenteric revascularization. The incidence of delirium after this surgery was estimated as high as 33% (36). We speculated that I/R attacks during surgery might serve as a risk factor for clinical postoperative delirium. Previous rat model acquiring ischemic attacks by clamping SMA for 90 min (12) resulted in high immediate mortality. Based on the evidence that intermittent ischemia exaggerated reperfusion injury in the small intestines (17), we improved the I/R protocol by applying repeated (three cycles) and transient (10/5 min for one I/R cycle) I/R procedures. The current model successfully developed behavioral changes associated with postoperative delirium, including anxiety and impaired cognition, while avoiding animal deaths during the experiments.

Regarding the specific behavioral tests, we found that intestinal I/R surgery disturbed the mice mainly in the Open Field and Y Maze tests, but not in the Buried Food Test. Compared to the sham mice, the I/R-injured mice showing less rearing time and less time spent in center region postoperatively indicated increased anxiety after surgery. These results capitulated the features of delirious patients who were agitated or restless after abdominal surgery. Meanwhile, the I/R-injured mice also showed the cognitive dysfunction at postoperative 24 h as evidenced in the Y Maze Test. This phenotype is consistent with the cognitive deficits in working memory and orientation observed in patients with delirium. I/R injury did not cause any significant changes in the Food Buried Test. The test was adopted according to Peng’s report (15) that can be used to assess postoperative delirium. However, in most cases the test was used to evaluate olfactory dysfunction, which was not a typical symptom associated surgery, and only to a lesser extent with delirium related inattention. Other behavior tests should be considered to comprehensively assess delirium-like phenotypes in the rodent models. Finally, given the multiple manifestations of delirium-like phenotype, the composite score encompassing different aspects of the assessment can serve as a comprehensive and comparable parameter to evaluate delirium among different animal groups. We adopted Peng’s tool (15), which calculates composite Z score based on a battery of behavior tests and is analogous to the formulation of CAM/CAM-ICU and CAM-Severity (37). We found that the current model caused ascending composite Z scores over 24 h after surgery, mimicking the natural course of clinical delirium. The current model thus represents a simple and clinically relevant mouse model of postoperative delirium.

One of the major findings of the current study is that postoperatively abnormal BLA activities may underpin the pathogenesis of the delirious phenotype. Microglia can respond rapidly to environmental changes and then adapt/maladapt to such changes by modulating neuronal activities with various microglia-derived molecules (4). Indeed, after I/R surgery, microglia were rapidly reactivated in various brain regions involved in emotion and cognition. Two regions, namely BLA and CA3, showed sustained microglia reactivation over 24 h. Remarkably, BLA was the only region in which the percentage of reactivated microglia was negatively correlated with cognitive deficits. We then applied MINO, recombinant IL-6 protein and IL-6 neutralizing antibody to manipulate microglia activities and found that microglia modulation in BLA was associated with changes in I/R-induced cognitive impairment, further supporting the BLA involvement. Previous studies identified IDO-1 as a critical modulator of depression- and anxiety-like behaviors induced by systemic inflammation (38, 39). IDO-1 is a rate-limiting metabolic enzyme of the kynurenine pathway composed of several neuroactive metabolites including QUIN, an endogenous *N*-methyl-D-aspartate receptor (NMDAR) agonist implicated in depression and cognitive deficits (6, 40). Although IDO-1 is expressed in different types of glial cells, microglia dominate the production of the neuroexcitatory metabolite QUIN in response to inflammatory mediators (41). These studies suggest that microglial IDO-1 could produce QUIN to modulate neural activities in the CNS. In this study, we found that IDO-1 and QUIN levels were significantly increased along with microglia reactivation in BLA after I/R surgery, while these changes were reduced by MINO administration, suggesting that abnormal microglia activation can lead to excessive QUIN in BLA, bringing about cognitive impairment after surgery. Previous studies have reported hippocampus or cortex were two activated regions after intestinal I/R injury (12–14). For example, Zhou et al. (12) reported activated microglia in the cortex and CA1 of hippocampus in rats after 1-h ischemia and 48-h perfusion of small intestine surgery. Hovens et al. (13, 14) screened the brain regions tagged with reactivated microglia after intestinal I/R surgery, including cortex, hippocampus, and BLA. However, since those results were obtained 1 week after surgery, the models should relate to delayed neurocognitive recovery rather than delirium. The finding in our study that abnormalities in the BLA started early in the disease deserves close attention. BLA is responsible for promoting the fear response and consolidating the cued fear memory (29). In a systematic review summarizing the qualitative findings of patients’ experiences of delirium, fear was an overarching feeling reported by the vast majority of patients (42). The results of our animal model were consistent with this clinical manifestation that implied alleviating patient’s fear as potentially effective therapies to reduce postoperative delirium.

I/R attacks induced a dramatic increase in IL-6, IL -1β, and TNF-α in the small intestines. However, along the gut-blood-brain axis only IL-6 was increased, but not IL-1β or TNF-α. Previous study showed that peripheral IL-6 can cross BBB (43). The findings that systemic administration of recombinant IL-6 protein and anti-IL-6 antibody increased and decreased IL-6 levels in BLA, respectively, supported the direct penetration of IL-6 after I/R attacks. A recent study using a similar rat model of intestinal I/R reported that plasma IL-6 was elevated over 24 h after surgery (13). In addition, two large clinical cohort studies reported that plasma IL-6 were increased in delirious patients shortly after surgery (44, 45) and IL-6 levels were significantly associated with an increased risk of postoperative delirium. However, the mechanism of how peripheral IL-6 mediated the brain disorder was not studied in these studies. In our study, microglia were considered as target cells of increased IL-6 since the IL-6 receptor is mainly detected in microglia in the CNS (46). Furthermore, IL-6 manipulation can enhance or reverse intestinal I/R-induced microglia reactivation supports microglia as potential targets of IL-6. In the meantime, it has been reported that IDO-1 expression could be upregulated by IL-6 in the brain (47, 48). We found that administration of IL-6 antibodies caused decreased IL-6 levels in the BLA accompanied by suppression of IDO-1/QUIN (41) which improved the cognitive function, while injection of IL-6 protein had opposite effects. These results provided evidence that intestinal I/R-induced IL-6 might penetrate BBB and derived the pathogenesis of postoperative delirium through activation of microglial IDO-1/QUIN metabolic pathway in the BLA. Our results together with previous evidence can serve as a pre-clinical justification for IL-6 modulation as a strategy to alleviate delirium associated cognitive impairment.

The current study had several limitations. First, younger adult mice were used in the study which did not represent the older people who have postoperative delirium more commonly. However, a great number of younger adult patients also develop postoperative delirium at an incidence from 5 to 13.9% (49). We expected the older mice would present grave delirium-like phenotype due to the high-risk factor of advanced age for delirium. Second, we did not evaluate the model in female mice, which did not reflect the same incidence of delirium in male and female patients. Future comparative studies need to be conducted to explore the difference in pathology between different age groups or sexes. Finally, the connection between microglial QUIN and BLA neuron activities was not thoroughly explored in the current study. The selective interference of microglial IDO-1 or neuronal NMDAR to validate the implication of the IDO-1/QUIN/NMDAR axis-dependent microglia-neuron interaction in the pathogenesis of postoperative delirium will be investigated to consolidate the findings of current study.

In conclusion, the repeated I/R insults on the SMA capitulated certain features of postoperative delirium. The core symptom of cognition decline was explained by peripheral IL-6-mediated microglial reactivation followed by IDO-1/QUIN production in the BLA. The model is a useful tool for delineating the mechanisms of delirium after abdominal surgery.

## Data availability statement

The original contributions presented in this study are included in the article/Supplementary material, further inquiries can be directed to the corresponding author.

## Ethics statement

The animal study was reviewed and approved by the Animal Experiment and Welfare Committee of Nanfang Hospital, Southern Medical University.

## Author contributions

J-LM, X-DL, and K-XL conceived and designed the study and prepared the manuscript, with editing and revision by all authors. J-LM, Y-HD, S-DQ, Y-YF, and FZ performed the experiments. J-LM, Y-HD, and Y-YF analyzed the data. All authors contributed to the article and approved the submitted version.
