# Supplementary material for: Peripheral interleukin-6-associated microglial QUIN elevation in basolateral amygdala contributed to cognitive dysfunction in a mouse model of postoperative delirium
Source: Front Med (Lausanne). 2022 Sep 9;9:998397. doi: 10.3389/fmed.2022.998397 (PMC9500157; doi:10.3389/fmed.2022.998397)
Supplement: Supplementary file 1 [file Table_1.doc]

**Supplementary Table 1.** The scoring system to grade histologic injury of small intestine

| Condition | Score | Description |
| --- | --- | --- |
| Mucosal injury | 0  1  2  3  4  5 | Normal mucosa  Subepithelial space (Gruenhagen’s) at villus tip  Moderate lifting of epithelium  Severe lifting of epithelium  Denuding of villi with exposed lamina propria  Disintegration of lamina propria with ulceration |
| Inflammation | 0  1  2  3  4  5 | Absent  Focal increase in lamina propria  Diffuse increase in lamina propria  Focal subendothelial collections  Diffuse subendothelial collections  Massive collections |
| Hyperemia/Hemorrhage | 0  1  2  3  4  5 | Absent  Dilated capillaries in lamina propria  Focal hemorrhage in lamina propria  Diffuse hemorrhage in lamina propria  Subendothelial hemorrhage  Massive hemorrhage |
