# Supplementary material for: Peripheral interleukin-6-associated microglial QUIN elevation in basolateral amygdala contributed to cognitive dysfunction in a mouse model of postoperative delirium
Source: Front Med (Lausanne). 2022 Sep 9;9:998397. doi: 10.3389/fmed.2022.998397 (PMC9500157; doi:10.3389/fmed.2022.998397)
Supplement: Supplementary file 2 [file Table_2.doc]

**Supplementary Table 2.** Total distance detected by OFT

Data shown as mean±SD. OFT: Open Field Test; I/R: ischemia and reperfusion; hrs: hours

| Time  Group | Baseline for 6hrs | 6 hrs | Baseline for 9hrs | 9 hrs | Baseline for 24hrs | 24 hrs |
| --- | --- | --- | --- | --- | --- | --- |
| Sham | 1695±77.79 | 940.4±73.78 | 1865±86.25 | 1009±77.10 | 1807±77.03 | 1122±81.82 |
| Repeated I/R | 1610±196.7 | 938.3±69.80 | 1869±96.21 | 952.4±79.71 | 1703±59.57 | 1158±75.39 |
