# Supplementary material for: Peripheral interleukin-6-associated microglial QUIN elevation in basolateral amygdala contributed to cognitive dysfunction in a mouse model of postoperative delirium
Source: Front Med (Lausanne). 2022 Sep 9;9:998397. doi: 10.3389/fmed.2022.998397 (PMC9500157; doi:10.3389/fmed.2022.998397)
Supplement: Supplementary file 3 [file Table_3.docx]

**Supplementary Table 3.** Total arm visits at Y maze

| Time Group | Baseline for 6hrs | 6 hrs | Baseline for 9hrs | 9 hrs | Baseline for 24hrs | 24 hrs |
| --- | --- | --- | --- | --- | --- | --- |
| Sham | 8.78±1.41 | 8.50±1.32 | 9.00±1.82 | 6.50±1.25 | 9.25±1.41 | 9.00±1.41 |
| Repeated I/R | 9.92±1.05 | 8.00±1.44 | 8.89±1.30 | 6.00±1.40 | 9.35±1.45 | 8.90±1.38 |

Data shown as mean±SD. I/R: ischemia and reperfusion; hrs: hours
